# Determination of extracellular matrix collagen fibril architectures and pathological remodeling by polarization dependent second harmonic microscopy

**DOI:** 10.1038/s41598-017-12398-0

**Published:** 2017-09-22

**Authors:** Denis Rouède, Emmanuel Schaub, Jean-Jacques Bellanger, Frédéric Ezan, Jean-Claude Scimeca, Georges Baffet, François Tiaho

**Affiliations:** 1 CNRS, Institut de Physique de Rennes, Département Matière molle, UMR UR1-CNRS 6251, Université de Rennes1, F-35042 Rennes, France; 2 INSERM, Laboratoire Traitement du Signal et de l’Image, UMR UR1-INSERM U642, Université de Rennes1, F-35042 Rennes, France; 3 INSERM, UMR1085, IRSET Institut de Recherche sur la Santé l’Environnement et le Travail, SFR Biosit, Université de Rennes1, F-35043 Rennes, France; 4 CNRS, INSERM, Université de Nice Sophia Antipolis, iBV, Nice, 06100 France

## Abstract

Polarization dependence second harmonic generation (P-SHG) microscopy is gaining increase popularity for *in situ* quantification of fibrillar protein architectures. In this report, we combine P-SHG microscopy, new linear least square (LLS) fitting and modeling to determine and convert the complex second-order non-linear optical anisotropy parameter ρ of several collagen rich tissues into a simple geometric organization of collagen fibrils. Modeling integrates a priori knowledge of polyhelical organization of collagen molecule polymers forming fibrils and bundles of fibrils as well as Poisson photonic shot noise of the detection system. The results, which accurately predict the known sub-microscopic hierarchical organization of collagen fibrils in several tissues, suggest that they can be subdivided into three classes according to their microscopic and macroscopic hierarchical organization of collagen fibrils. They also show, for the first time to our knowledge, intrahepatic spatial discrimination between genuine fibrotic and non-fibrotic vessels. CCl_4_-treated livers are characterized by an increase in the percentage of fibrotic vessels and their remodeling involves peri-portal compaction and alignment of collagen fibrils that should contribute to portal hypertension. This integrated P-SHG image analysis method is a powerful tool that should open new avenue for the determination of pathophysiological and chemo-mechanical cues impacting collagen fibrils organization.

## Introduction

Collagens play a central role in the formation of fibrils networks involved in the architecture of tissues and organs. In extracellular matrixes (ECM), the physical compressive and tensile strains generated by cell traction are key mechanisms involved in the long-range ordering and remodeling of collagen fibrils^1,2^. These fibrils, consisting of long and filamentous polymers of collagen molecules, are arrays of axial and lateral supramolecular assembly of quarter-staggered collagen molecules resulting in 67 nm periodic striation observed at ultrastructural level with transmission electron microscopy. While much is known about the different hierarchical level of fibrillogenesis, comparatively little is known about how collagen fibrils assemble together into the diverse supramolecular arrangements found in the body. Moreover, remodeling of collagen fibrils involved in several pathologies encompassing fibrosis, cancer, bone and several connective tissues diseases also awaits a precise 3D description.

Label-free second harmonic generation (SHG) process relies on a nonlinear optical interaction with hyperpolarizable non centrosymetric endogenous fibrillar proteins like collagen and myosin causing scattered coherent radiation at twice the fundamental frequency^3,4^. Thus, it has proved to be an extremely beneficial contrast mechanism for label-free imaging of these endogenous molecules *in situ*, *in vivo*, in physiological as well as in disease state. Polarization dependence second harmonic generation (P-SHG) microscopy that enables quantification of organizational changes in fibrillar protein is gaining increase popularity for investigating fibrillar collagen-rich tissues with the desire to extract as much structural information as possible^5^. SHG has been successfully used for the assessment of liver fibrosis against histological gold standard Fibrosis-Metavir/Ishak staging of patients with moderate to severe fibrosis/cirrhoris^6,7^. However, none of these studies have evaluated ECM fibrillar collagen remodeling despite several reports showing that fibroblast-induced mechanical forces mediate structural rearrangements of ECM collagen fibrils^1,8^. Interestingly, assessing ECM remodeling may prove useful for evaluating the response to anti-fibrotic treatment and fibrosis regression both in human and experimental animal models. To address the issue of ECM collagen remodeling, we use in this study the classical CCl_4_ fibrotic mouse liver model in which fibrosis scoring is difficult due to the absence of well-defined scores like the Metavir. Nevertheless, several histologic and biochemical similarities has been long established between chronically CCl_4_-treated animals and human liver fibrosis/cirrhosis^9–11^.

We focus our study on collagen fibrils expansion and remodeling around portal tracts and central veins, which are the site of fibrosis initiation in F1 Metavir stage of liver fibrosis^12^. To achieve our goal, we extend our recently reported simple and fast linear least square (LLS) fitting method^13^, that enables processing P-SHG images at pixel-resolution level, in order to determine fibrils orientations, values of second-order nonlinear optical susceptibility tensor coefficients and anisotropy parameter *ρ* that is usually described as a marker of fibril organization^14,15^. Then, we convert *ρ* values of several collagen rich tissues into a simple geometric organization of collagen fibrils integrating a priori knowledge of hierarchical organization of collagen molecule assembly into fibrils and bundle of fibrils as well as Poisson photonic shot noise of the detectors.

Our first result was to validate the modeling approach by retrieving the known polyhelical arrangement of fibrillar proteins (collagen and myosin) in tendon, skin and striated muscle. We next analyzed a great number of tissues resulting in the identification of three main classes based on macroscopic fibrillar orientation, polyhelical organization and microscopic fibrillar disorder. Finally, we showed that liver fibrosis characterized by fibrillar remodeling results in reduced fibrillar disorder.

Altogether, the results show that P-SHG images analysis with LLS fitting method is a powerful method for estimating *in situ* fibrillar collagen organization in physiological and in disease tissues.

## Material and Methods

### Preparation of biological samples

Experiments were performed with rat and mouse tissues. All animal procedures were approved by local health care committees CREEA R-2012-GB-01 (Rennes, France), CIEPAL NCE/2012-69 (Nice, France). They were performed in accordance with European Community guidelines for the care and use of laboratory animals (Directive 2010/63/UE). Adult Wistar rats (200–300 g) were euthanized by CO_2_ inhalation. Collagen-rich and muscle tissues were dissected, fixed over night with 4% paraformaldehyde (PFA) in phosphate buffer saline at 4 °C, and rinsed at least three times with PBS. For SHG imaging, dissected pieces (100–200 μm thickness) of collagen-rich and muscle tissues were mounted in 50% glycerol-PBS solution and stabilized between two coverslips sealed with nail polish. 4–5 µm thick sections of bone samples stained with Hematoxylin Eosin Saffron (HES) were prepared using standard protocols encompassing 24 h fixation, 4 weeks decalcification, paraffin embedment. Wild-type C57 black6 mice received intraperitoneal injection of either 0,5 µg CCl_4_ (Sigma, St Louis, MO) dissolved in oil in order to induce liver fibrosis or the olive oil solvent for control mice. Injections were performed 3 times (D1, D3 and D7) the first week followed by single injection every week lasting 8 weeks. Mice were euthanized at D1 or at W10, livers were harvested and fixed in 4% paraformaldehyde. They were named oil-D1 and oil-W8 for control oil-injected and W8-CCl_4_ for CCl_4_-injected mice based on the type and duration of the injections. Each hepatic vessel of both oil- and CCl_4_-injected mice were further sub-classified in “group1” or “group2” respectively for non-fibrotic and fibrotic vessels based on SHG-imaging properties of each vessel. For SHG-imaging, 10 μm thick sections of liver slices mounted between microscope glass slides and coverslips were obtained from the Biosit-H2P2 core (*histopathologie*.*univ-rennes1*.*fr/)* according to established standard procedure. All rats and mice were cared for in accordance to the “Guide for the Care and Use of Laboratory Animals” (Directive 2010/63/UE): animal care facility approval number A06-088-14; rat testing approved protocol NCE/2012-69; mouse testing approved protocol NCA 2008–02.

### Image acquisition and analysis

Images are acquired on a custom made SHG microscope based on an inverted microscope (IX71, Olympus, Japan). The laser source is a tunable IR 80 MHz femtosecond Ti:Sa laser (MAITAI, Spectra Physics). The polarization of the beam is controlled by an achromatic half wave plate and an achromatic quarter wave plate mounted on motorized rotary stages (PR50CC, Newport). High NA water immersion objective (Olympus LUMFL 60 W × 1.1 NA) (Olympus, Tokyo, Japan) was used for applying 10–20 mW of 740 nm excitation at the sample (PSF is about 0.4 × 1.2 μm). SHG signal was collected in forward direction using high NA objective (Olympus, LUMFl 60XW, NA = 1.1). The SHG signal is detected using high sensitivity single photon GasAsP photomultipliers (H7421–40, Hamamatsu). The photo-pulses are counted by counters of our general purpose acquisition module (NI-USB 6363, National Instruments). The acquisition software was developed with LabView 2010, and in C (Visual Studio 2010, Microsoft). It is important to note that in addition to tissue clearing with 50% glycerol treatment all polarization dependent SHG images were acquired at a depth < 20 μm from the tissue surface to minimize depolarization^16^ and birefringence effect^17,18^. Image analysis and simulations were performed using MATLAB (MathWorks, Natick, MA, USA). All experimental P-SHG polarization stacks were obtained for input polarization angles *α* uniformly distributed between 0° and 180° with 20° increments. This incremental step is a good sampling compromise for accurate acquisition of SHG parameters and quality of the recording, avoiding motion artifact. Data were obtained from at least 3 samples in each experimental condition and at least 4 ROIs per sample. Automatic thresholding was performed on the polarization stacks before fitting with LLS method. The total image processing time for a 512 × 512 pixels image is about 3 s, that includes stack reading, image thresholding, LLS computing time and image display. The LLS computing time is about 20% of the total. For mice liver pathological collagen remodeling, Student t-test statistical analyses were performed on Oil-D1 (3 animals), Oil-W8 (8 animals) and CCl_4_-injected (8 animals) mice from respectively 32, 101 and 105 liver vessels. We note p/oil-D1 and p/oil-W8, p-values obtained versus respectively oil-D1 and oil-W8 control vessels.

### Strategy to calculate *ρ*_exp_ and *ρ*_poiss_

**Figure 1:**
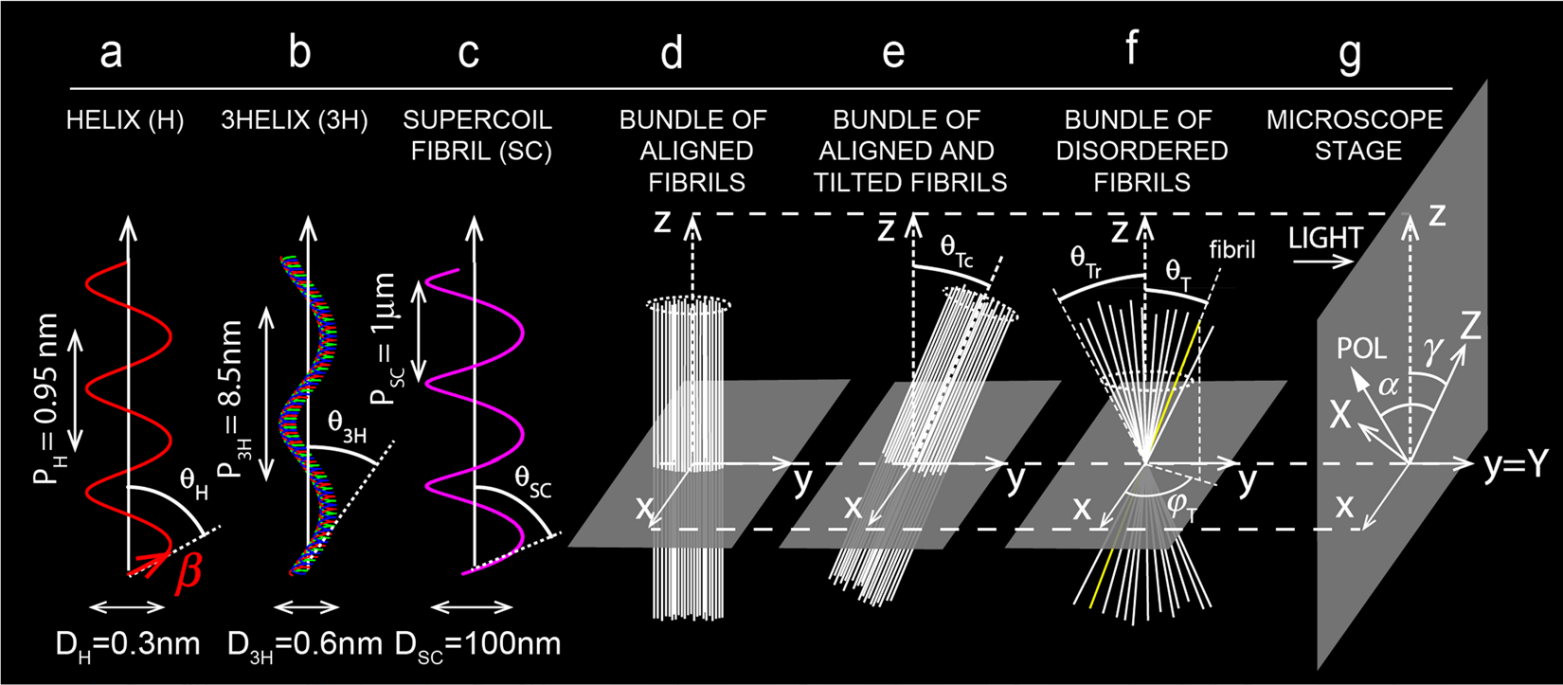
Hierarchical organization of collagen fibrils. (**a**) Cylindrical organization of peptide bonds β around single helix (H) main axis. Helix angle θ_H_ = 53°, pitch P_H_ = 0.95 nm and diameter D_H_ = 0.3 nm. (**b**) Cylindrical coil-coil of three single helices around triple helix (3helix, 3 H) main axis. 3helix angle ${\theta }_{3H}=12^\circ$, pitch P_3H_ = 8.5 nm and diameter D_3H_ = 0.6 nm. (**c**) Cylindrical organization of the triple helix around main axis of the supercoiled (SC) fibril. Supercoil angle ${\theta }_{SC}=17^\circ$, pitch P_SC_ = 1 μm and diameter D_SC_ = 100 nm. (**d**) Bundle of well aligned fibrils with bundle main axis along z direction that is in the plane of the microscope stage (see Fig. 1g). (**e**) Bundle of well aligned and tilted fibrils along y direction with angle ${\theta }_{Tc}$ with z direction. (**f**) Bundle of disordered fibrils within a cone with half apex angle ${\theta }_{Tr}$ with z direction. (**g**) Microscope stage with fixed laboratory coordinates system X, Y, Z. $\gamma$ and $\alpha$ are angles between respectively z direction and input polarization with Z direction. Light propagates in y = Y direction. For (**a**–**c**), diameters D and pitches P are from references^23,24^.

Macroscopic second-order nonlinear optical susceptibility tensor *χ* ^(2)^ is obtained from macroscopic second-order nonlinear optical susceptibility tensor of individual fibril ${\chi }_{f}^{(2)}$ taking into account the contribution of all the N_f_ fibrils within the focusing volume. ${\chi }_{f}^{(2)}$ was calculated for the case of polyhelical organization of collagen molecules considered here (see Fig. 1a–c). We found that only two independent components ${\chi }_{33}^{f}$ and ${\chi }_{31}^{f}={\chi }_{15}^{f}$ of ${\chi }_{f}^{(2)}$, when written in coordinates system x, y, z (see Fig. 1g), mainly contribute to SHG signal under the assumption of only one tensor element *β* in microscopic molecular hyperpolarizability tensor ${\beta }^{(2)}$. Analytical expressions of ${\chi }_{33}^{f}$ and ${\chi }_{31}^{f}$ were obtained as a function of ${\theta }_{H},\,{\theta }_{3H},\,{\theta }_{SC}$ for case 1 (Fig. 1d), and of the tilted angle ${\theta }_{Tc}$ for case 2 (Fig. 1e) or the pair of disordered angles ${\theta }_{T},\,\,{\phi }_{T}$ for case 3 (Fig. 1f). Corresponding components ${\chi }_{33}$ and ${\chi }_{31}$ of *χ* ^(2)^ were derived from ${\chi }_{33}^{f}$ and ${\chi }_{31}^{f}$ by summing contributions of all the N_f_ fibrils within the focusing volume. Theoretical anisotropy parameter ${\rho }_{th}={\chi }_{33}/{\chi }_{31}$ was deduced.

On the other hand, P-SHG intensity is given by the usual expression^19^

$${I}^{2\omega }\propto {[\sin 2(\alpha -\gamma )]}^{2}+{[{\sin }^{2}(\alpha -\gamma )+\rho {\cos }^{2}(\alpha -\gamma )]}^{2}\,,$$

where $\gamma$ and $\alpha$ are angles between respectively z direction and input polarization with fixed Z direction of the microscope stage (Fig. 1e). In order to deduce the tissular architectural organization of collagen fibrils for each pixel of a P-SHG image, experimental anisotropy parameter *ρ*_exp_, obtained by fitting of ${I}^{2\omega }$, has to be compared to theoretical one ${\rho }_{th}$, calculated for an a priori fibrillar organization. Moreover, we have recently shown that experimental anisotropy parameter *ρ*_exp_ is highly dependent of Poisson photonic shot noise of the detectors^13^, and it is of paramount importance to take it into account in the calculation of ${\rho }_{th}$ when compared to *ρ*_exp_ Briefly, P-SHG intensity ${I}^{2\omega }$ is firstly generated for a particular set of $\alpha$ and *γ* angles and at given value of ${\rho }_{th}$, assuming a priori fibrillar organization. It is secondly rendered noisy after having added Poisson photonic shot noise and fitted using LLS method, so as to deduce Poisson noise anisotropy parameter *ρ*_poiss_. In summary, three anisotropy parameters ${\rho }_{\exp }$, ${\rho }_{th}$ and *ρ*_poiss_ are determined for each pixel of a P-SHG image. *ρ*_exp_ is obtained by LLS fitting of experimental P-SHG data, ${\rho }_{th}$ and *ρ*_poiss_ are calculated from a particular fibrillar organization respectively without and with taking account of Poisson photonic shot noise. Moreover, correlation between number of fibrils and P-SHG intensity for each pixel of the P-SHG image is obtained by assuming an inverse quadratic law ${N}_{f}\propto \sqrt{{I}^{2\omega }}$ in agreement with a second-order nonlinear optical process.

### Theory

Second harmonic electric fields ${{\boldsymbol{E}}}^{2\omega }$ emitted at 2ω originate from nonlinear polarization ${{\boldsymbol{E}}}^{2\omega }={\chi }^{(2)}{{\boldsymbol{E}}}^{\omega }{{\boldsymbol{E}}}^{\omega }$ induced by mixing of intense electric fields ${{\boldsymbol{E}}}^{\omega }$ at ω in a medium characterized by a macroscopic second-order nonlinear optical susceptibility tensor *χ* ^(2)^. As a rule *χ* ^(2)^ is derived from associated microscopic molecular hyperpolarizability tensor ${\beta }^{(2)}$, and throughout the rest of the document, we make the assumption that the elementary molecular sources of SHG signal in collagenous and myosin tissues are the peptide bonds along the collagen and myosin single helix scaffold with only one non-zero tensor element ${\beta }_{333}=\beta$ ^14,19–21^. In order to calculate nonlinear optical susceptibility *χ* ^(2)^ and anisotropy parameter $\rho$, organization of peptide bonds *β* must be specified.

At microscopic level, mechanism of fibril formation is well known^22^, it is a three step processes starting from a single helix polypeptide chain with helix polar angle ${\theta }_{H}$ (Fig. 1a) that coils into a stable triple helix with polar angle ${\theta }_{3H}$ (Fig. 1b) by association of three polypeptide chains called tropocollagen representing the collagen molecule^22^. For collagen, three single helices (3 H) are needed to form the collagen molecule with coil-coil angle of about ${\theta }_{3H}=12^\circ$ and for myosin, two single helices (2 H) are needed to form the myosin molecule with coil-coil angle of about ${\theta }_{2H}=12^\circ$ ^23^. Tropocollagen units assemble in a quarter-staggered array to form straight fibrils as in tendon. A supercoiled process (Fig. 1c) is taken into account in dermis where triple helical tropocollagen is twisted at a constant polar angle ${\theta }_{SC}$ to form supercoiled micro fibrils or fibrils with an axis different from that of the tropocollagen molecule^22^.

At macroscopic level, collagen fibrillar organization can be complex and specific for each collagen tissue. Three simple cases of fibrillar organization are considered in the following. Case 1 (Fig. 1d) describes a bundle of well-aligned fibrils with bundle main axis along z direction that is in the plane of the microscope stage (see Fig. 1g). This case describes straight fibrils as in tendon. Case 2 (Fig. 1e) describes well-aligned fibrils that are tilted (out of plane) along y direction at constant tilt angle ${\theta }_{Tc}$ with z direction; y direction corresponds to main direction of light propagation (see Fig. 1g). In soft tissues like liver, additional fibrillar disorder has to be taken into account to better-fit experimental data. Case 3 (Fig. 1f) illustrates a simple case of disorder with random distribution of fibrils within a cone with half apex angle ${\theta }_{Tr}$ with z direction. Each fibril is defined by a couple of random azimuthal ${\phi }_{T}\in (0,2\pi )$ and polar ${\theta }_{T}\in (0,{\theta }_{Tr})$ angles.

Our strategy to calculate nonlinear optical susceptibility *χ* ^(2)^ and corresponding anisotropy parameter $\rho$ that is a marker of fibril organization is described in Material and Methods. In summary, two anisotropy parameters *ρ*_exp_ and *ρ*_poiss_ are determined for each pixel of a P-SHG image. *ρ*_exp_ is obtained by LLS fitting of experimental P-SHG data and *ρ*_poiss_ is calculated from a particular fibrillar organization $({\theta }_{H},\,\,{\theta }_{3H},\,\,{\theta }_{SC},\,\,{\theta }_{T})$ taking account of Poisson photonic shot noise. Correlation coefficient ${r}_{k}$ defined as ${r}_{k}=1-{d}_{k}$, minimizing the Kolmogorov-Smirnov distance d_k_^25^ between the two empirical cumulative distribution functions associated to *ρ*_exp_ and *ρ*_poiss_ is used to determine the best correspondence between *ρ*_poiss_ and *ρ*_exp_ data for any particular ROI of the P-SHG image. ${r}_{k}$ varies from 1 for two ideally identical cumulate distribution functions to 0 when the two series of values lie in two distinct intervals.

## Experimental Results

### P-SHG image analysis from rat EDL tendon, rat skin and rat EDL muscle

**Figure 2:**
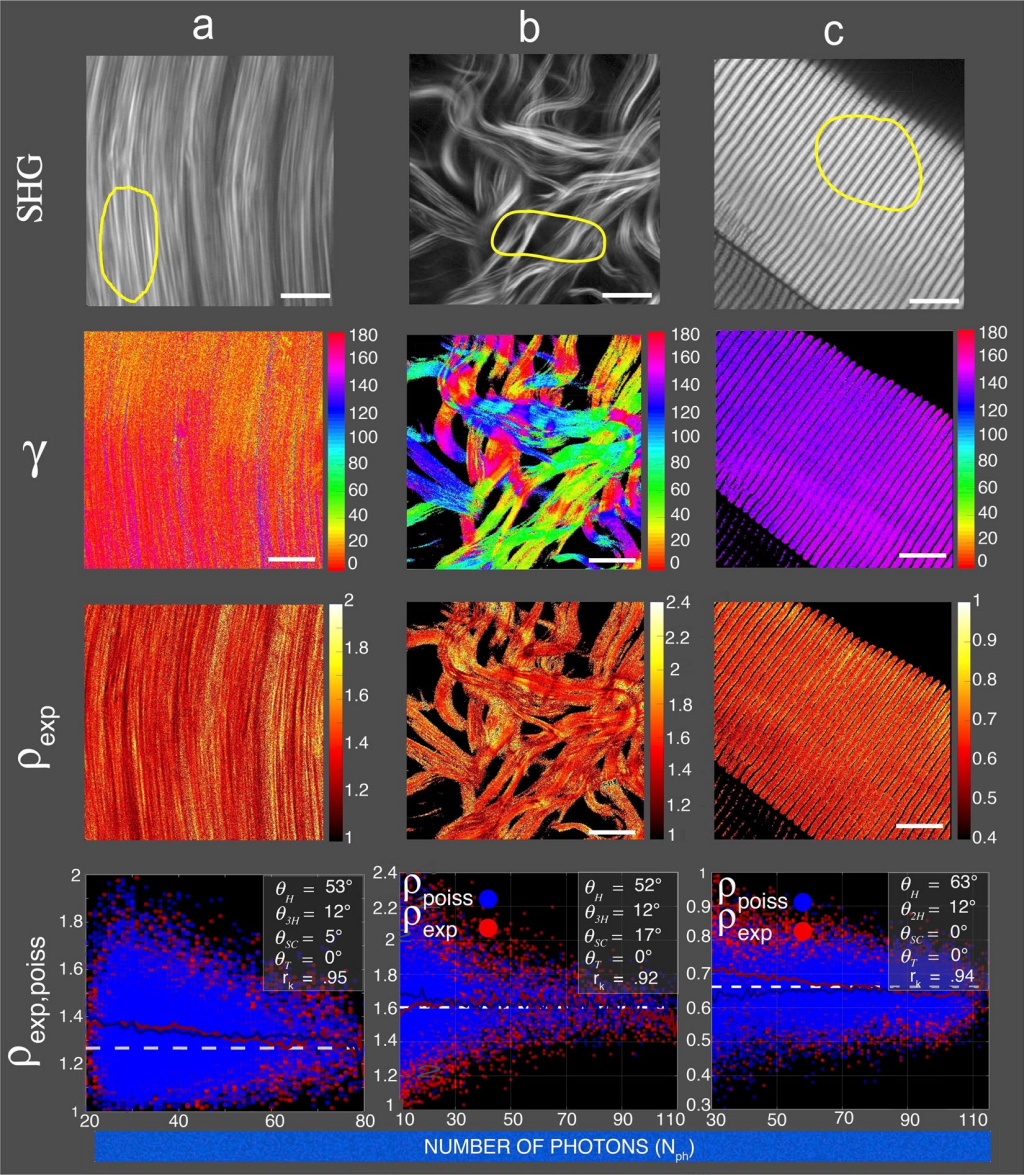
P-SHG image analysis from different tissues. (**a**) rat EDL tendon, (**b**) rat skin and (**c**) rat EDL muscle. The first row represents typical 512 × 512 pixels SHG images obtained by averaging the polarization stack. Note that these images correspond to the square root of the SHG intensities. The second row is the angular fibrillar orientation map *γ* for each pixel of the SHG images. The scale is in degrees and is indicated by the right vertical color bar. Note that direction for 0° is north. The third row corresponds to the 2D distribution of *ρ*_exp_ for each pixel of the SHG images. The last row represents the distribution of *ρ*_exp_ (red dots) and *ρ*_poiss_ (blue dots) as a function of the P-SHG stack mean photons number per pixel N_ph_ for the ROI indicated in the SHG images (first row). Their corresponding mean values are full lines in respectively red and blue colors. Theoretical mean values ${\rho }_{th}$ without Poisson noise are indicated by the horizontal white dotted lines. The best correlation coefficient r_k_ between ${\rho }_{\exp }$ and *ρ*_poiss_ is obtained for values of ${\theta }_{H}$, ${\theta }_{3H}$, ${\theta }_{SC}$ and ${\theta }_{T}$ angles that are written in the inset of each figure. Scale bars are 10 μm.

In order to validate our integrated P-SHG image analysis method, we choose tissues with well-known fibrillar organization. Thus, the results correlating *ρ*_exp_ and *ρ*_poiss_ and the underlying hierarchical organization angles are shown for rat extensor digitorum longus tendon, rat skin and rat extensor digitorum longus muscle in Fig. 2. These tissues were chosen as typical examples of respectively straight coiled collagen fibrils, supercoiled collagen fibrils^22^ and straight coiled myosin fibrils^26^. For these tissues, SHG image (1st row), pixel-resolved fibrillar orientation (2nd row), map of ${\rho }_{\exp }$ (3rd row) and dispersion of *ρ*_exp_ and of *ρ*_poiss_ (4th row) are shown as a function of the P-SHG stack mean photons number per pixel N_ph_. Macroscopically, we consistently obtained quasi-homogeneous values of *γ* in the overall field of view for tendons (Fig. 2a) and muscles (Fig. 2c) while heterogeneous values are observed for skin tissues (Fig. 2b). Microscopically, correlation of *ρ*_exp_ and *ρ*_poiss_ (Fig. 2, last row), was realized by superposition of five plots as follows. Red and blue dots represent respectively *ρ*_exp_ and *ρ*_poiss_, continuous red and blue lines are their corresponding mean values and white dotted line represents ${\rho }_{th}$. For these three tissues, the best fit was obtained for ${\theta }_{T}=0^\circ$ indicating well-aligned fibrils within each pixel of the SHG image. Results show a good agreement between *ρ*_exp_ and *ρ*_poiss_ values taking into account both their known hierarchical organization and Poisson noise. *ρ*_exp_ values are 1.3 ± 0.09, 1.6 ± 0.14 and 0.6 ± 0.07, respectively for tendon, skin and muscle tissues. Theoretical parameters maximizing r_k_, resulting in the best correlation between *ρ*_exp_ and *ρ*_poiss_ for each tissue, are indicated in the inset of the last row. For tendon collagen ${\theta }_{H}=52.7^\circ \pm 0.4^\circ$(N = 18) and $\,{\theta }_{3H}=12^\circ$. For skin collagen, ${\theta }_{H}=52.0^\circ \pm 0.3^\circ$ (N = 18),$\,{\theta }_{3H}=12^\circ$ and ${\theta }_{SC}=17^\circ$. For muscle myosin ${\theta }_{H}=62.6^\circ \pm 0.4^\circ$ (N = 18) and ${\theta }_{3H}=12^\circ .$ Note that r_k_ was found to be greater than 0.9 for the proposed fibrillar organization of these three tissues.

Altogether these results indicate that at sub-micrometric level (pixel level), all three tissues are characterized by well-aligned fibrils. This order is conserved at macroscopic level for tendon and striated muscle. However for skin, supra-pixel disordered has to be taken into account. Moreover, our proposed hierarchical organization deduced from P-SHG analysis is fully validated due to the good agreement between our results and those reported using SEM or X-ray diffraction methods^22,23^.

### P-SHG image analysis from different classes of collagen rich tissues

**Figure 3:**
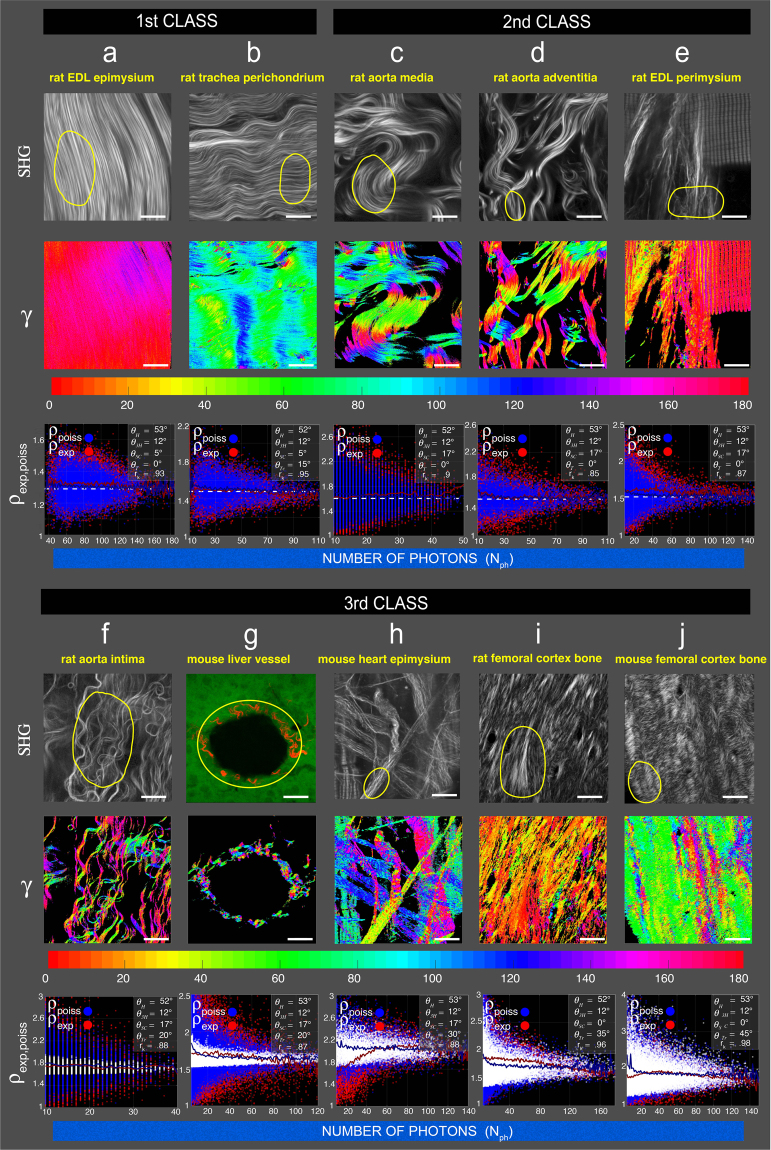
Classification of different collagen rich tissues based on P-SHG analysis. The first class corresponds to (**a**) rat EDL epimysium and (**b**) rat tracheal perichondrium. This class is characterized by preponderant two helical levels of organization ${\theta }_{H},{\theta }_{3H}$ found in straight fibrils. Note that for rat tracheal perichondrium, constant tilt ${\theta }_{Tc}$ has been added in the simulation to better-fit experimental data (see Fig. 1e). This is justified by the fact that undulations are clearly visible on the SHG image. The second class includes (**c**) rat aorta media, (**d**) rat aorta adventitia and (**e**) rat EDL perimysium. It is characterized by three helical levels of organization ${\theta }_{H},{\theta }_{3H},{\theta }_{SC}$. The third class encompasses (**f**) rat aorta intima, (**g**) liver vessel, (**h**) mousse heart epimysium, (**i**) rat femoral cortex bone (longitudinal section) and (**j**) mouse femoral cortex bone (transverse section). This latter class can be differentiated from the formers by significant disorder of collagen fibrils necessitating an additional level of fibrillar disorder ${\theta }_{Tr}$. Color-coded plots of the last row have the same meaning as in Fig. 2. Scale bars are 20 μm.

We next extend in Fig. 3 the pixel-resolved fibril organization to different collagen rich tissues. At submicrometric level, the results show that these tissues can be divided in 3 classes, straight coiled well-aligned fibrils (class 1), supercoiled well-aligned fibrils (class 2) and dis-aligned fibrils (class 3) based on the estimated ${\theta }_{H}$, ${\theta }_{3H}$, ${\theta }_{SC}$ and ${\theta }_{Tr}$ angles. For each tissue, a preferred hierarchical organization of collagen fibril is proposed for the indicated ROIs.

Class 1 (Fig. 3a and b) encompasses rat extensor digitorum lungus epimysium and rat tracheal perichondrium. Mean *ρ*_exp_ values are 1.3–1.4 for the indicated ROI in the SHG image. r_k_ > 0.9, accurately estimates the preponderant two helical levels ${\theta }_{H}=53^\circ ,{\theta }_{3H}=12^\circ$ involved in the formation of triple helices tropocollagen molecules.

Class 2 (Fig. 3c–e) includes rat aorta media, rat aorta adventitia and rat extensor digitorum lungus perimysium. Mean *ρ*_exp_ values are 1.6–1.7 for the indicated ROI in the SHG image and r_k_ > 0.85 indicates that the three-levels of hierarchical organization are ${\theta }_{H}=53^\circ ,$ ${\theta }_{3H}=12^\circ$ and ${\theta }_{SC}=17^\circ$ corresponding to supercoiled tropocollagen molecules.

Class 3 (Fig. 3f–j) is represented by rat aorta intima, mouse liver vessel, mouse cardiac epimysium, rat femoral cortex bone (longitudinal section), mouse femoral cortex bone (transverse section). Mean *ρ*_exp_ values are 1.6–1.9 for the indicated ROI in the SHG image. In contrast to classes 1 and 2, an additional fibrillar disorder ${\theta }_{Tr}$ was necessary to achieve good correlation coefficient r_k_ > 0.8. The main feature of this third class is that, because of fibrillar dis-alignment ${\theta }_{Tr}\ne 0$, ${\rho }_{th}$ values (white dots) present a drastic fluctuation around a mean value and is no longer a constant straight white dotted line as for class 1 and 2. Hence, for mouse cardiac epimysium, rat and mouse femoral cortex bones, dispersion of ${\rho }_{th}$ values due to collagen fibril disorder is of similar magnitude as dispersion of *ρ*_poiss_ values.

Macroscopically (supra-pixel level), the first class of collagen-rich tissue, like tendon collagen fibril, is also characterized by less dispersion of fibrils orientation *γ* values (Fig. 3a,b, 2^rd^ row) showing that fibrils tend to be well-ordered at all hierarchical levels. Values of fibrils orientation *γ* for second and third classes are clearly more disperse, indicated drastic fibrils disorder at macroscopic level.

Finally, the features of the three classes of collagen rich-tissues can be summarized as follow. The first class has microscopic and macroscopic well-aligned fibrils. The second class has microscopic well aligned but macroscopic dis-aligned fibrils. The third class has microscopic and macroscopic dis-aligned fibrils. Altogether, these results show that pixel-resolved 3D sub-micrometric organization of collagen fibrils can be well estimated by correlating experimental *ρ*_exp_ and theoretical *ρ*_poiss_ values taking into account a priori fibrils hierarchical organization without (classes 1,2) or with (class 3) fibrillar disorder, considering ubiquitous Poisson noise.

### P-SHG image analysis of control and fibrotic mouse liver vessels

**Figure 4:**
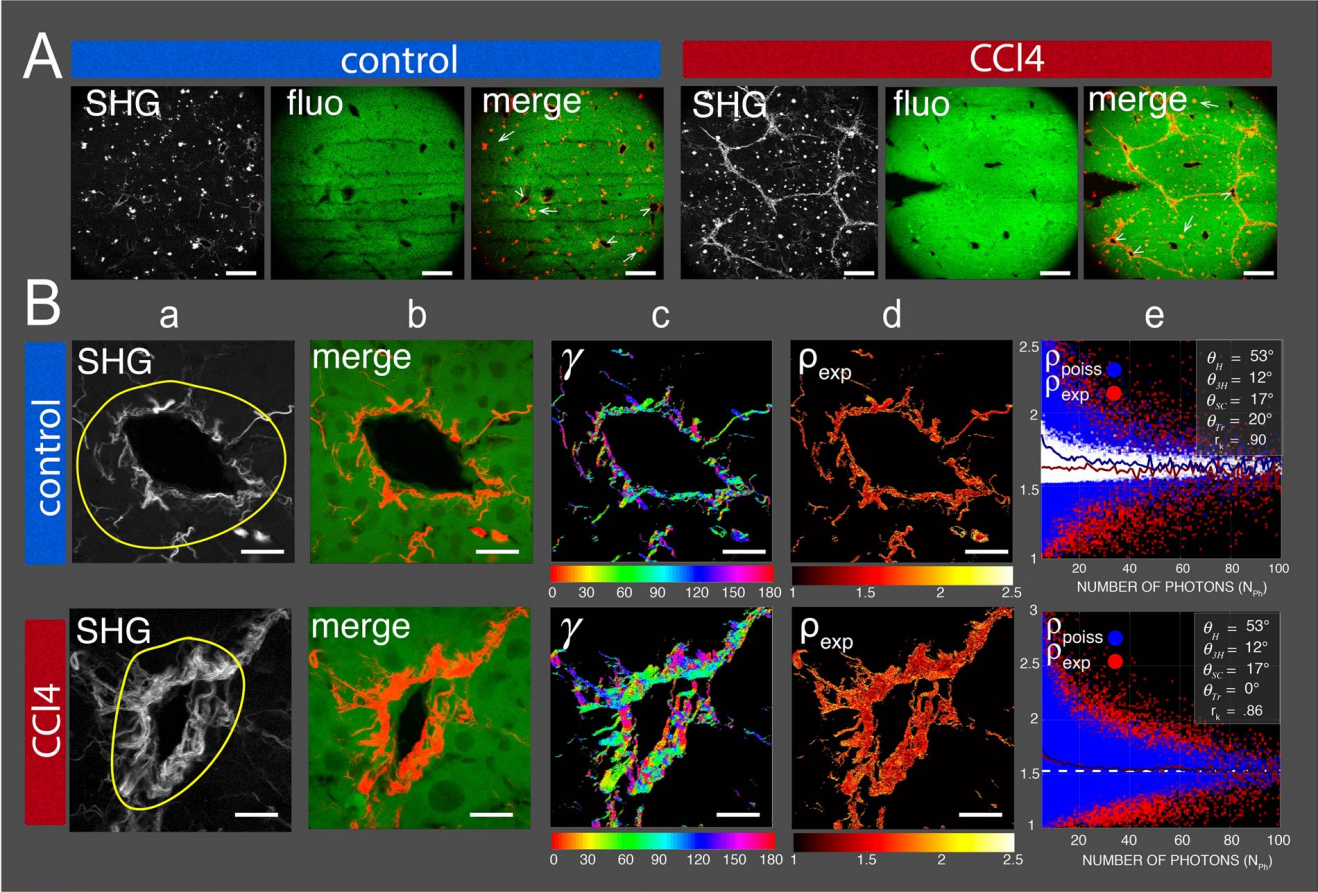
SHG and fluorescence images analysis of control and fibrotic mouse livers. (**A**) SHG (average of polarization stacks images), autofluorescent and merge images of control and CCl_4_ treated mice livers taken at lower magnification (10X). Note the presence of vascular (arrowheads) and granular (arrows) collagen in both control and CCl_4_ treated livers (see merge). Note also that fibrillar collagen between vessels is a characteristic feature of fibrotic livers of CCl_4_ treated mice. Scale bars are 200 μm. (**B**) P-SHG image analysis of collagen fibrils organization in control and fibrotic mouse liver vessels taken at higher magnification (60X). First and second rows represent respectively control and CCl_4_ treated mice vessels. Column (a) represents typical SHG images of control and CCl_4_ treated mice vessels. Column (b) is the merge of autofluorescent and SHG images. Column (c) is the pixel-resolved angular orientation *γ* of fibrils in degrees indicated by the bottom horizontal color bar. Note that direction for 0° is north. Column (d) corresponds to the 2D distribution of *ρ*_exp_ for each pixel of the SHG images. Column (e) represents the distribution of *ρ*_exp_ and *ρ*_poiss_ and their means as a function of the P-SHG stack mean photons number per pixel N_ph_ for the ROI indicated in the SHG image (first column). Color-coded plots have the same meaning as in Fig. 2. Scale bars are 20 μm.

We then tested the power of our integrated P-SHG image analysis method to discriminate *in situ* the organization of collagen fibrils between control and fibrotic liver vessels. Repetitive CCl_4_ intoxication for several weeks results in chronic hepatic injury leading to liver fibrosis/cirrhosis in many animals including mouse^9^. As mentioned in material and methods section, mice fibrotic livers were obtained after 8 weeks administration with CCl_4_ dissolved in olive oil. Extracellular matrix collagen fibrils accumulation and organization were determined by SHG/TPEF imaging and the results are shown at low (10X objective, Fig. 4A and Supplementary fig. 1) and high magnifications (60X objective, Fig. 4B and Supplementary fig. 2). The main feature of SHG images of livers from CCl_4_-injected mice compared to control mice (injected with solvent olive oil) is the development of bridging fibrosis characterized by a great number of septa with collagen fibrils connecting adjacent vessels (portals and central vessels) as illustrated in Fig. 4A at low magnification (10X objective). Common to both control and CCl_4_ treated livers, vascular (arrowheads) and granular (arrows) collagen are also present as illustrated by the merge images.

For control and CCl_4_ treated mice liver vessels, SHG merge of SHG and autofluorescent images, maps of *γ* and *ρ*_exp_ are shown in Fig. 4B(a–d). At macroscopic level, collagen fibrils orientations *γ* are clearly widely disperse for both control and CCl_4_-treated vessels indicating a similar great fibrils disorder (see Fig. 4B(c)). Distributions of *ρ*_exp_ and *ρ*_poiss_, that are plotted as a function of the P-SHG stack mean photons number per pixel N_ph_ for the ROI indicated in the SHG image, are shown in Fig. 4B(e). Results indicate that mean values of *ρ*_exp_ and *ρ*_poiss_ coincide at high photon level to ${\rho }_{th}=1.6$ and ${\rho }_{th}=1.5$ for respectively control and CCl_4_ treated mice liver vessels. Whereas the best set of hierarchical angles ${\theta }_{H}=53^\circ$,$\,{\theta }_{3H}=12^\circ$, ${\theta }_{SC}=17^\circ$ are similar for both tissues, disorder level was found to be respectively ${\theta }_{Tr}=20^\circ$ and ${\theta }_{Tr}=0^\circ$ (see insets of Fig. 4B(e)).

**Figure 5:**
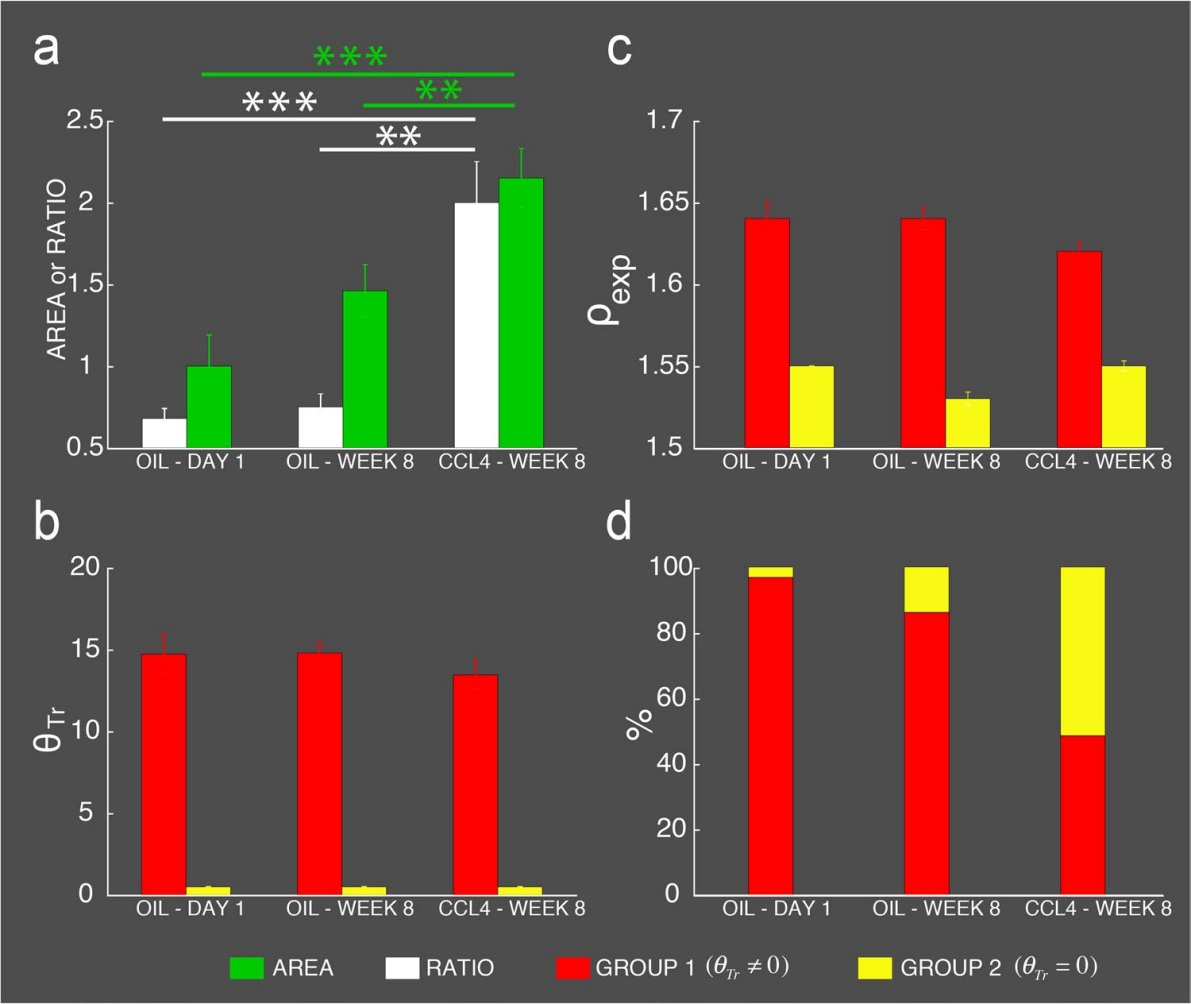
Impact of oil and CCl_4_ injections on liver portal tracts collagen fibrils expansion and remodeling after one-day oil injection (oil-D1), 8 weeks oil injection (Oil-W8) and 8 weeks CCl_4_ injection (W8-CCl_4_). (**a**) Collagen proportionate area (green bars) and ratio of peri-portal collagen area over luminal area (white bars). Note that for collagen proportionate area normalization relative to the mean area obtained for one-day oil injection was undertaken. (**b**) Collagen fibril angular disorder ${\theta }_{Tr}$ determined the sub-classification of the vessels in group 1 (red bars, ${\theta }_{Tr} > 0$) and group 2 (yellow bars, ${\theta }_{Tr}=0$). Note that for group 2 vessels, an arbitrary value of ${\theta }_{Tr}=0.5$ is plotted for clarity. (**c**) Experimental mean anisotropy parameter *ρ*_exp_ after sub-classification of the vessels in group 1 (red bars, ${\theta }_{Tr}\ne 0$) and group 2 (yellow bars, ${\theta }_{Tr}=0$) based on ${\theta }_{Tr}$. (**d**) Percentage of group 1 (red bars) and group 2 (yellow bars) vessels as a function of the indicated experimental condition. The numbers (n) of vessels are 32, 101 and 105 for respectively Oil-D1 (3 animals), Oil-W8 (8 animals) and CCl4-injected (8 animals) mice. Note that the legend of the bars is located at the bottom of the figure. Note also that two (**) and three asterisks (***) have respectively the following significant meaning 0.001 < p < 0.01, p < 0.001 based on student t-test.

Comparison of P-SHG images features of portal tracts collagen fibrils between control and CCl_4_ injected livers (illustrated in Fig. 4B), are quantified in Fig. 5 and Table 1 for one-day oil injected (Oil-D1, n = 32 vessels from 3 animals), 8 weeks oil-injected (Oil-W8, n = 101 vessels from 8 animals) and 8 weeks CCl_4_-injected (n = 105 vessels from 8 animals) mice.

**Table 1:** Statistical *ρ*_exp_ and ${\theta }_{Tr}$ values in degrees obtained from liver vessels of one-day oil injected (Oil-D1), 8 weeks oil-injected (Oil-W8) and 8 weeks CCl_4_-injected mice, for $5\le {N}_{ph}\le 50$. Note that p / Oil-D1 and p / Oil-W8 are p-values obtained versus respectively Oil-D1 and Oil-W8 controls.

	*ρ*_exp_			θ_Tr_		
	Oil-D1	Oil-W8	CCl_4_-W8	Oil-D1	Oil-W8	CCl_4_-W8
Mean	1.64	1.62	1.58	14.22	12.91	6.86
NB	32	101	105	32	101	105
SEM	0.01	< 0.01	< 0.01	1.23	0.70	0.80
p/Oil-D1		0.25	< 0.001		0.43	< 0.001
p/Oil-W8			< 0.001			< 0.001

For peri-portal collagen area, a significant increase (p < 0.01) of about 115% and 50% are found in CCl_4_-treated livers compared to respectively controls Oil-D1and Oil-W8 (Fig. 5a, green bars). This collagen expansion is accompanied by a significant increase (p < 0.01) in the ratio of peri-portal collagen area over portal luminal area (Fig. 5a, white bars).

For anisotropy parameter *ρ*_exp_, a significant reduction (p < 0.001) from 1.62 ± 0.01 in control (Oil-W8) to 1.58 ± 0.01 in CCl_4_-treated livers was observed for $5\le {N}_{ph}\le 50$ (Table 1). This interval was chosen because of the great difference observed between mean values of *ρ*_exp_ and ${\rho }_{poiss}$, suggesting additional disorder not taken into account in the proposed hierarchical organization. *ρ*_exp_ values were similar (1.64 ± 0.01 versus 1.62 ± 0.01) for both controls Oil-D1 and Oil-W8 (Table 1). As mentioned above, control livers vessels belong to the third class of collagen rich tissue characterized by microscopic and macroscopic collagen fibrils disordered. Thus, the mean *ρ*_exp_ value of about 1.62 (for Oil-W8) is indicative of microscopic uniform fibril disorder with an average ${\theta }_{Tr}=13^\circ$ (Table 1). However, for CCl_4_-treated liver vessels, the mean *ρ*_exp_ value of 1.58 is indicative of significant (p < 0.001) reduced fibril disorder at microscopic level with an average ${\theta }_{Tr}=7^\circ$ (Table 1). A close examination of ${\theta }_{Tr}$ values for both control and CCl_4_-treated mice portal vessels indicate that they can be sub-classified in two groups ${\theta }_{Tr}\ne 0$ and ${\theta }_{Tr}=0$ (Fig. 5b, red and yellows bars). The portal vessels of the first group have the following features $10^\circ  < {\theta }_{Tr} < 25^\circ$ (Fig. 5b, red bars), ${\rho }_{\exp } > 1.6$ (Fig. 5c, red bars) and peri-portal collagen area is smaller than luminal area (Fig. 5a, white bars). In contrast, portal vessels of group 2 have well ordered portal collagen fibrils with ${\theta }_{Tr}=0$ (Fig. 5b, yellow bars), pixel-resolved ${\rho }_{\exp } < 1.6$ (Fig. 5c, yellow bars) and luminal area smaller than peri-portal collagen area (Fig. 5a, white bars). Based on this classification the percentage of group 2 portal vessels ${\theta }_{Tr}=0$ was found to increase from 3% in Oil-D1 livers to 51% in CCl_4_-treated livers (Fig. 5d, yellow bars).

Altogether, these results indicate that P-SHG is a powerful tool for intrahepatic sub-discrimination between genuine fibrotic (group2) from non fibrotic (group1) vessels in both control and CCl_4_-treated mice. The later livers are characterized by an increase in the percentage of fibrotic vessels and a parallel decrease of non fibrotic vessels compared to control livers. Thus, in addition to peri-portal collagen accumulation, genuine fibrotic vessels collagen fibrils are remodeled and become well-ordered at microscopic level ${\theta }_{Tr}=0$.

## Discussion

We have extended and developed new LLS fitting analysis to gain insight into the specific sub-micrometric and macroscopic hierarchical molecular organization of collagen and myosin fibrils from different tissues. The present results show that combined P-SHG and LLS method is a powerful tool for estimating the *in situ* fibrillar collagen organization in physiological and disease tissues. We have previously shown that LLS method is accurate and powerful for pixel-by-pixel analysis of nonlinear susceptibility ${\chi }^{(2)}$ coefficients and fibril orientation. This method is very easy to perform for non-expert in numerical signal processing^13^. In the present report, LLS analysis was extended to retrieve a priori pixel-resolved hierarchical molecular organization. Thus, for a given ROI, the sub-microscopic hierarchical organization of fibrillar proteins^22,23^ can be retrieved by correlating the distribution of experimental anisotropic parameters *ρ*_exp_ to calculated ones *ρ*_poiss_, taking account of Poisson photonic shot noise of the detection system. Moreover, the results indicate that at sub-micrometric level (pixel-resolved level), tendon, skin and striated muscle are characterized by bundles of well-aligned fibrils. This ordering is conserved at macroscopic level for tendon and striated muscle but for skin supra-pixel disordered (large dispersion of *γ* has to be taken into account.

In this study, we have analyzed a great number of collagen-rich tissues, resulting in the identification of three main classes based on microscopic parameters, *ρ*_exp_, polyhelical organization angles ${\theta }_{H},{\theta }_{3H},{\theta }_{SC}$, fibrillar disorder ${\theta }_{Tr}$ and macroscopic fibrillar orientation $\gamma$. The first class encompasses tendon, muscular epimysium and tracheal perichondrium. It has microscopic and macroscopic well-aligned straight fibrils with preponderant two levels of fibrillar hierarchical organization ${\theta }_{H}=53^\circ ,{\theta }_{3H}=12^\circ$. The second class includes vascular media/adventitia and muscular perimysium. It is characterized by three hierarchical levels of fibrillar hierarchical organization ${\theta }_{H}=53^\circ ,{\theta }_{3H}=12^\circ$ and ${\theta }_{SC}=17^\circ$. At microscopic level, fibrils are well ordered ${\theta }_{Tr}=0^\circ$. However, at macroscopic level, fibrils are dis-aligned with more disperse $\gamma$ values. The third class encompasses aorta intima, liver vessel, heart epimysium and femoral cortex bone. Compared to the previous classes, it has an additional level of microscopic fibrillar disorder ${\theta }_{Tr}\ne 0^\circ$. Like the second class, it has also more disperse $\gamma$ values. Our classification is further supported by the good agreement found between our proposed hierarchical organization and that reported using SEM microscopy for perichondrium, aorta and cardiac epimysium^27,28^. For bones, SHG image, fibrils orientation $\gamma$, *ρ*_exp_ and *ρ*_poiss_ are in good agreement with the proposed twisted plywood model of lamellar organization^29,30^. Furthermore, at microscopic level, pixel-resolved *ρ*_exp_ values suggest that several lamellae with different angular orientations are found within a single excitation volume in agreement with recent results from human femoral bone using dual focused ion beam scanning electron microscopy^31^.

The structural architecture of these three classes might reflect their tissular function. Collagen fibrils orientations $\gamma$ might functionally reflect directions of traction and compression forces exerted by fibroblasts or myofibroblasts on ECM^32,33^. Sub-microscopic and macroscopic straight-coiled and well-aligned fibrils in tendon enables higher Young modulus to sustained parallel forces arising during skeletal muscles contraction. Supercoiled well-aligned bundle of fibrils at sub-microscopic level is often found in soft tissue^22^. In vascular aorta their presence in media and adventitia enables high resistance of vascular walls to both axial and transverse oriented mechanical pressure. Thus, supercoiled (twisted) fibrils are shaped to sustained multidirectional forces and compliance of soft tissues might be generated by the macroscopic dispersion of fibrils orientations $\gamma$ found in group 2 and 3 collagen tissues.

Tuer *et al*.^15^ have recently proposed a model that partially takes into account collagen fibrils hierarchical organization for interpreting P-SHG data of biological tissues. The main improvement provided by our model is that it integrates a direct correlation between experimental anisotropy parameter *ρ*_exp_ and theoretical one *ρ*_poiss_ calculated from a priori fibrillar organization integrating the background Poisson photonic shot noise of the detection system. Therefore, our model enables better assessment of fibrils disorder that often has a large contribution to low photons SHG signal and can be misestimate through signal thresholding. This has been highlighted with the particular case of collagen fibrillar organization of liver tissues. A second advantage of our model is that it is an extension of our previous LLS method^13^. Thus, the estimation of experimental anisotropy parameter *ρ*_exp_ is accurate, fast and does not need more sophisticated microscope setup with output polarizers like for polarization in polarization out (PIPO) method resulting in time consuming for both experimental acquisition design and non linear least square analysis methods.

From the results of our study, the first question that comes to mind is what potential interest does P-SHG has with regard to several other methodologies routinely used to quantify liver fibrosis (for review see^34^). The gold standard histological activity index method commonly used by clinicians and hepatopathologists for scoring, grading and staging chronic hepatitis is based on the staining of histological sections by Masson trichrome and picrosirius red dyes. Picrosirius red specificity for fibrillar collagen can be greatly increased by exploiting its property of exalting the birefringence of the latter by polarized light microscopy^35^. These methods rely on pathological examination of liver biopsies by a trained pathologist for numerical scoring of cellular necrosis, tissue inflammation, fibrosis and alteration of its architecture, thus, providing a semi-quantitative assessment of the observed histological features of liver biopsies. Histological methods are often associated to computer-assisted digital optical image analysis for determination of collagen proportionate area (CPA). Since CPA is a continuous variable, it represents a better quantitative histological index of liver fibrosis that is clinically correlated to hepatic venous pressure gradient, which is considered a good predictor of survival and liver decompensation in patients with cirrhosis. Subjective intra-observer and inter-observer variability of the scoring is the main disadvantage of these histological techniques. More recently, it has been shown that second harmonic generation signal, provides extremely beneficial contrast mechanism for label-free specific imaging of endogenous fibrillar collagen. SHG signal enables robust and objective digital image analysis measurement of CPA that correlates with the ‘Fibrosis’-METAVIR staging^6,7^. While SHG imaging is the most sensitive and specific method for measurement of CPA, it does not say anything about the three-dimensional organization of the collagen fibrils that underlie and sustained the extra- and intra-hepatic mechanical forces arising in fibrotic liver. Thus, none of the previous studies have evaluated ECM fibrillar collagen remodeling occurring during hepatic fibrosis progression despite several reports showing that fibroblasts and myofibroblasts can generate mechanical forces affecting structural rearrangements of ECM collagen fibrils^1,2,8,32^. We address this issue, using the classical CCl_4_ fibrotic mouse liver model. We focus our study on collagen fibrils remodeling occurring around portal tracts and central veins, which are the early sites of fibrosis in F1 Metavir stage of liver fibrosis^12^. We report for the first time, the rearrangement of vascular collagen fibrils occurring during ECM remodeling. The results show that fibrotic portal tracts and central veins can be distinguished with greater sensibility from non-fibrotic vessels based on pixel-resolved ${\theta }_{Tr}$ values. At submicroscopic level, non-fibrotic vessels have the following features. Collagen fibrils are disordered $10^\circ  < {\theta }_{Tr} < 25^\circ$, ${\rho }_{\exp } > 1.6$ and portal luminal area is often greater than peri-portal collagen area. This latter characteristic is often observed in vein vessels. In contrast, fibrotic vessels have well ordered portal collagen fibrils ${\theta }_{Tr}=0^\circ$, ${\rho }_{\exp } < 1.6$ and luminal area is smaller than peri-portal collagen area. This latter property being a general property of arterial vessels that resist higher pressure, and consequently have thicker media and adventitia. Thus, the striking result is that, in addition to peri-portal collagen accumulation, fibrotic vessels collagen fibrils are remodeled and become well-ordered at microscopic level. This indicates that liver fibrosis is characterized by a progressive shift of portal collagen fibrils architecture from class 3 to class 2 like in dermis, aorta media, aorta adventitia and skeletal muscle perimisyum. Thus fibrosis commutes the peri-portal vein architecture to an arterial one. It is tempting to suggest that the driving force of this collagen remodeling is the blood pressure. Interestingly, a relationship between porto-pulmonary hypertension and lung fibrosis has recently been established in a mouse model of CCl_4_-mediated cirrhosis^36^ suggesting that the heterogeneity of peri-portal tracts and central veins collagen remodeling that we found in CCl_4_-treated liver could reflect heterogeneity in the amount of portal tracts and central vein hypertension. According to action-reaction mechanical law, myofiboblasts should contract in response to an increase in blood pressure resulting in perivascular collagen fibrils compression and reduction in dispersion of their angular orientations. Interestingly, autocrine and endothelin-dependent myofibroblast contraction has been shown in human cirrhotic livers^37^. This remodeling of the collagen fibrils will result in an increase in stiffness of vascular wall and a subsequent rise in intra-portal tracts and central veins tone as already suggested^37^. Thus, a positive feed forward loop sustaining a rise in vascular pressure should be generated, leading to more differentiated myofibroblasts and expansion of fibrosis. This speculative mechanism deserves to be tested in experimental conditions of controlled blood pressure in order to determine the precise role of mechanical stresses in the development of liver fibrosis. In the hypothesis of validation of this scenario, we would have at our disposal a means for estimating the amount of pressure in each individual vessel through their precise remodeling using P-SHG imaging. Therefore, this study is a first step towards understanding the correlation of the organization of fibrillar collagen with the mechanical forces that underlie the pathophysiology of chronic liver diseases. Moreover, assessing ECM remodeling may prove useful for evaluating the response to anti-fibrotic treatment and fibrosis regression both in human and experimental animal models.

## Conclusion

This study highlights the power of combined P-SHG microscopy and LLS fitting method to retrieve pixel-resolved sub-microscopic hierarchical organization of fibrillar proteins by correlating experimental second-order non-linear optical anisotropy parameter ${\rho }_{\exp }$ to theoretical one *ρ*_poiss_ taking into account the background Poisson photonic shot noise of the detectors.

Our study of several collagen-rich tissues enables the identification of three main classes based on polyhelical organization of collagen polypeptide chains, fibrillar disorder and macroscopic fibrillar orientation. This classification is further supported by the good agreement found between our proposed hierarchical organization and that reported using SEM microscopy.

We show, for the first time that, in addition to peri-vascular collagen fibrils accumulation in portal tracts and central veins, remodeling of fibrotic livers are characterized by a feed forward mechanism of peri-vascular collagen fibrils compaction and alignment that should contribute to portal hypertension.

Our results open new avenue for the determination of physical impacts on collagen fibrils organization and could greatly contribute to the identification of predictive factors conditioning liver fibrosis regression in experimental animal models and translational selection of patients predisposed to benefiting from new anti-fibrotic treatments.

## Electronic supplementary material

SHG and fluorescence images analysis of control and fibrotic mouse livers.
