# Supplementary material for: Determination of extracellular matrix collagen fibril architectures and pathological remodeling by polarization dependent second harmonic microscopy
Source: Sci Rep. 2017 Sep 22;7:12197. doi: 10.1038/s41598-017-12398-0 (PMC5610346; doi:10.1038/s41598-017-12398-0)
Supplement: Supplementary file 1 — SHG and fluorescence images analysis of control and fibrotic mouse livers. [file 41598_2017_12398_MOESM1_ESM.pdf]

SUPPLEMENTARY INFORMATION

Determination of extracellular matrix collagen fibril architectures and pathological remodeling by polarization dependent second harmonic microscopy

Denis Rouède,[1,*] Emmanuel Schaub,[1] Jean-Jacques Bellanger,[2] Frédéric Ezan,[3] Jean-Claude Scimeca,[4] Georges Baffet[3] and François Tiaho[3]

[1] CNRS, Institut de Physique de Rennes, Département Matière molle, UMR UR1-CNRS 6251, Université de Rennes1, F-35042 Rennes, France
[2] INSERM, Laboratoire Traitement du Signal et de l'Image, UMR UR1-INSERM U642, Université de Rennes1, F-35042 Rennes, France
[3] INSERM, UMR1085, IRSET Institut de Recherche sur la Santé l'Environnement et le Travail, SFR Biosit, Université de Rennes1, F-35043 Rennes, France
[4] Université Côte d'Azur, CNRS, Inserm, iBV, France
*francois.tiaho@univ-rennes1.fr

The PDF file includes

Supplementary Figure 1. Typical SHG, autofluorescent and merge images of control and CCl_4 treated mice livers taken at lower magnification (10X).

Supplementary Figure 2. P-SHG image analysis of collagen fibrils organization in control (Oil-D1 and Oil-W8) and fibrotic CCl_4 treated liver vessels taken at higher magnification (60X).

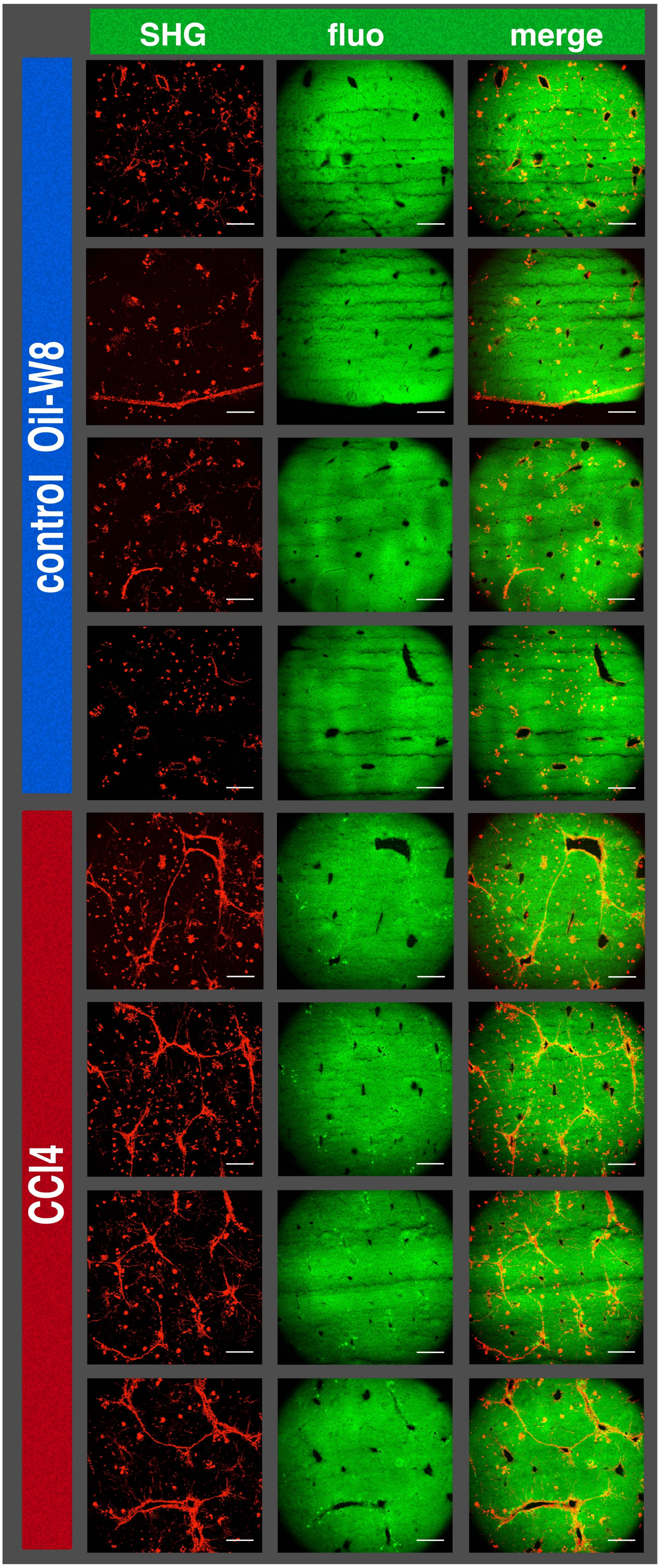

Supplementary Figure 1. Typical SHG, autofluorescent and merge images of control and CCl_4 treated mice livers taken at low magnification (10X). Scale bars are 200 µm.

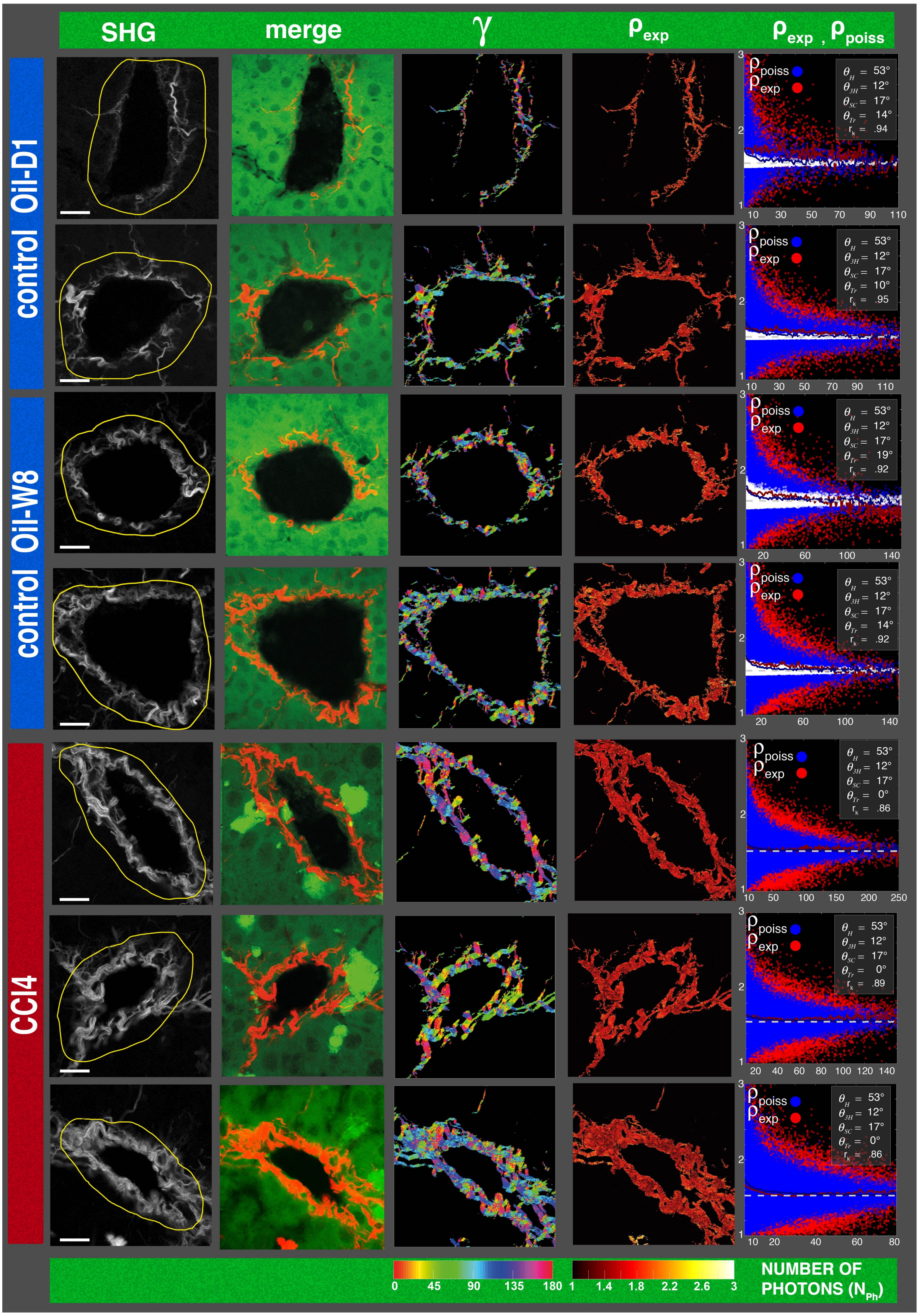

Supplementary Figure 2. P-SHG image analysis of collagen fibrils organization in control (Oil-D1 and Oil-W8) and fibrotic CCl_4 treated liver vessels taken at higher magnification (60X). First column (SHG) represents typical SHG images. Second column (merge) is the merge of autofluorescent and SHG images. Third column (γ) is the pixel-resolved angular orientation γ of fibrils in degrees indicated by the bottom horizontal color bar. Note that direction for 0° is north. Fourth column (ρ_{exp}) corresponds to the 2D distribution of ρ_{exp} for

each pixel of the SHG images. The scale is indicated by the bottom horizontal color bar. Fifth column ($\rho_{\exp}$, ρ_{poiss}) represents the distribution of $\rho_{\exp}$ (red dots) and ρ_{poiss} (blue dots) as a function of the P-SHG stack mean photons number per pixel N_{ph} for the ROI indicated in the SHG images (first column). Their corresponding mean values are full lines in respectively red and blue colors. Distribution of ρ_{th} and corresponding mean value without Poisson noise are indicated by respectively the white dots and the dotted line in gray color. The best correlation coefficient r_k between $\rho_{\exp}$ and ρ_{poiss} is obtained for values of θ_H, θ_{3H}, θ_{SC} and θ_{Tr} angles that are written in the inset of each figure. Scale bars are 20 µm.
